# Pyogenic Granuloma of the Sigmoid Colon causing Intussusception in an Infant 

**Published:** 2015-05-01

**Authors:** Salvatore Garofalo, Michael Mostert, Isabella Morra, Maria Grazia Cortese, Riccardo Guanà, Alessandro Mussa, Mario Canesi, Giulia Carbonaro, Alessia Cerrina, Luisa Ferrero, Davide Cussa, Jurgen Schleef

**Affiliations:** 1 Department of Paediatric Surgery, Regina Margherita Children's Hospital, Italy; 2 Department of Paediatrics, Regina Margherita Children's Hospital, Italy; 3 Department of Pathology, Regina Margherita Children's Hospital, Italy; 4 Department of Obstetrics and Gynaecology, Regina Margherita Children's Hospital, Italy

**Keywords:** Pyogenic granuloma, Progesterone, Pregnancy, Sigmoid tumor, Intussusception, Infant

## Abstract

Pyogenic granuloma is a benign vascular tumor that may affect the gastrointestinal tract. This report describes a rare case of sigmoid-colon pyogenic granuloma in a 4-month-old boy causing intussusception. Resection and anastomosis were curative. The mother had history of high dose of progesterone exposure during initial weeks of conception for vaginal bleeding. This may point towards etiology of the lesion.

## CASE REPORT

A 4-month-old boy was referred with sudden onset of colicky abdominal pain, vomiting, and rectal prolapse. Ultrasonic evaluation of the abdomen revealed intussusception. Contrast enema successfully reduced the intussusception and revealed a 2cm x1.9cm tumor-like lesion in the sigmoid colon that partially occluded the lumen (Fig. 1A). Routine blood tests were normal. Diagnostic single-port transumbilical laparoscopy was immediately performed which revealed a serosal tumefaction at the sigmoid colon level. Laparoscopy was converted to open surgery which confirmed the lesion. The colon was incised opposite to the mass revealing a polypoid, sessile, ulcerated tumor. Segmental resection-anastomosis of the sigmoid colon was performed. The post-operative course and the two years follow-up were uneventful.

**Figure:**
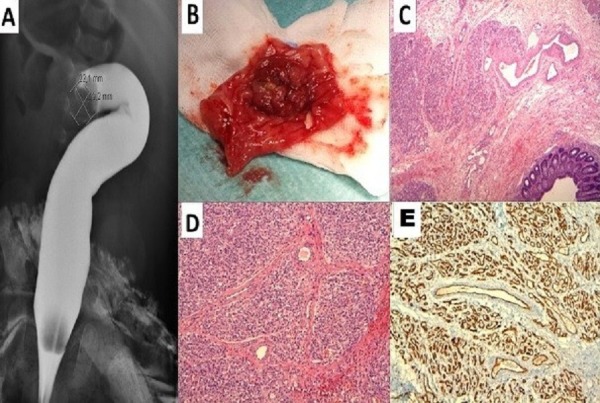
Figure 1: A: Diagnostic and therapeutic barium enema showing colocolic intussusception. B: Gross specimen of the sigmoid tract resected. C: Lobular hemangioma in the submucosa. D: Lobular growth of vessels. E: Immunostaining for CD 31 high-lights closely-packed capillaries.

Detailed obstetric history revealed that during initial weeks of pregnancy there was frequent vaginal bleeding that was treated with micronized progesterone vaginal ovule (dosed 200 mg) for nineteen weeks and a weekly intramuscular injection of 340 mg of 17-hydroxyprogesterone caproate (17-OHPC) for seventeen weeks (maintenance therapy). The child was born by cesarean section at 34th week of gestation following the onset of maternal hypertension and interruption of fetal growth.

Histopathological examination showed a submucosal lesion with ulcerated mucosal lining and consisted of a lobular hemangioma in a fibrous matrix (Fig. 1C, 1D). Antibodies against CD31 highlighted closely packed capillaries (Fig. 1E), while antibodies against estrogen receptors (SP1 Ventana) and progesterone receptors (1E2 Ventana) were negative.

## DISCUSSION

The etiology of pyogenic granuloma remains unclear. Supposed predisposing factors reported in literature are trauma, angiogenic stimulating factors, and hormones overgrowth as observed in pregnancy or in peripubertal age.[1] Hormonal imbalance during pregnancy associated with bacterial plaque and gingival inflammation was supposed to cause pyogenic granuloma on the oral mucosa. Generally, the lesion appears in the second or third month of pregnancy and disappears after the delivery.[2] Estrogen promotes granulation tissue formation and enhances macrophage production of vascular endothelial growth factor (VEGF), favoring the development of pyogenic granuloma in pregnancy.[3] Progesterone also promotes VEGF expression and enhances chronic tissue reaction in gingival tissue.[4] Both progesterone and estrogen inhibit granuloma cell apoptosis.[4]

17-OHPC is a very useful therapy in reducing preterm birth rates in women and is detectable in maternal and fetal blood for at least forty-four days after the last injection as micronized progesterone that also helps to limit preterm delivery.[5] The voluminous size of the granuloma in our case probably be explained referring to an early origin of the lesion in pregnancy, after some unidentified mucosal damages, and the subsequent early exogenous pharmacological stimulation. Prolonged and intense progesterone exposure in the first twenty-three weeks of pregnancy may have promoted the considerable growth of the polyp. Furthermore, in the absence of extracellular hormone stimulation (as it happens after birth) progesterone receptors degrade (half-life is just a few days), explaining receptor negativity at the histology.

Finally, according to Whitaker the quantity of estrogen or progesterone receptors in pyogenic granuloma is less important in the pathogenesis of this lesion than the circulating hormone levels.[6] This case is possibly another example of how diverse progesterone effects are, but further studies are required to support this hypothesis and to find hormonal markers to detect these lesions after birth. To conclude, pyogenic granuloma is a rare clinical entity that can act as a lead point in intestinal intussusception. Its association with full-dose hormonal supplementation during pregnancy could explain rapid growth of these lesions and possible complications. Women receiving these hormones during initial days of pregnancy must be educated about development of these complications in the baby after birth.

## Footnotes

**Source of Support:** Nil

**Conflict of Interest:** None declared

